# Expression of CD1a and Type-1 Polarization Are Dissociated in Human Monocyte-Derived Dendritic Cells

**DOI:** 10.1371/journal.pone.0140432

**Published:** 2015-10-13

**Authors:** Brigitta Mester, Evelyn Bauer, Catherine E. Wood, Ian F. Hermans, Olivier Gasser

**Affiliations:** 1 Malaghan Institute of Medical Research, Wellington, New Zealand; 2 Capital and Coast District Health Board, Wellington, New Zealand; 3 School of Biological Sciences, Victoria University of Wellington, Wellington, New Zealand; 4 Maurice Wilkins Centre, Wellington, New Zealand; Karolinska Institutet, SWEDEN

## Abstract

Ex vivo generated monocyte-derived dendritic cell (moDC)-vaccines have long been touted as promising immunotherapeutic agents for cancer treatment, although the response rate generally remains low. The reasons for this are still unclear and confounded by the diversity in manufacturing protocols that may affect moDC function. Preclinical studies have shown that the stimulatory function of dendritic cells can be improved by engaging invariant NKT cells in vivo through the presentation of the glycolipid alpha-galactosylceramide via CD1d. However, expression of CD1d on moDC has been shown to be negatively correlated with expression of CD1a, which in turn has been suggested to be a surrogate marker for IL-12 secreting type-1 polarized moDC, the preferred functional characteristics for cancer vaccines. Here we challenge this notion by showing that plasma-derived lipids drive functional levels of CD1d expression, while CD1a expression can vary considerably in these cells without being correlated with a loss of polarization or immunogenicity.

## Introduction

Targeting antigens to dendritic cells (DC) has been central to most immunotherapeutic strategies against cancer. A common approach is to load antigens onto autologous DC that have been generated from precursors ex vivo. Monocyte-derived (mo)DC are often used because they can be prepared in large numbers from human blood for clinical use, are capable of eliciting T cell responses in humans [1], and have a good safety profile [2, 3]. However, to date vaccine studies with moDC have failed to show sufficient benefit to have an impact on clinical practice [2, 3]. Although tumor-derived immunosuppression is likely to have played a major factor in reducing efficacy, there are also concerns that moDC vaccines are qualitatively heterogeneous, with a lack of comparability across studies.

In this context, the expression of CD1a has been associated with a type-1 polarized moDC phenotype, featuring significant capacity to manufacture IL-12 and potent immunogenicity in vitro, attributes that are favorable for vaccine generation [4, 5]. Conversely, lack of CD1a expression has been associated with a diminished capacity of moDC to produce IL-12 and an inability to polarize naïve CD4^+^ T cells to a Th1 phenotype. Importantly, the expression of CD1d seems to be inversely correlated with CD1a [6, 7], and by inference increased expression of CD1d would be associated with a non-desirable moDC phenotype. This is of relevance, as preclinical studies have shown that the stimulatory function of DC can be improved through interaction with CD1d-restricted NKT cells in vivo, a strategy achieved by loading the cells with the CD1d-binding glycolipid alpha-galactosylceramide (alpha-GalCer) or other NKT cell agonists before injection in mice or humans [8–11]. The functional dichotomy between CD1a and CD1d expression on moDC would therefore be a concern to exploit this activity, as a favorable type-1 polarized moDC phenotype is likely to have little to no expression of CD1d. Here we examined the phenotype and function of moDC generated from several healthy donors under different culture conditions to explore the relationship between CD1a and CD1d expression and cellular function.

## Results and Discussion

### Impact of autologous plasma on expression of CD1d, phenotype and function of moDC

**Fig 1:**
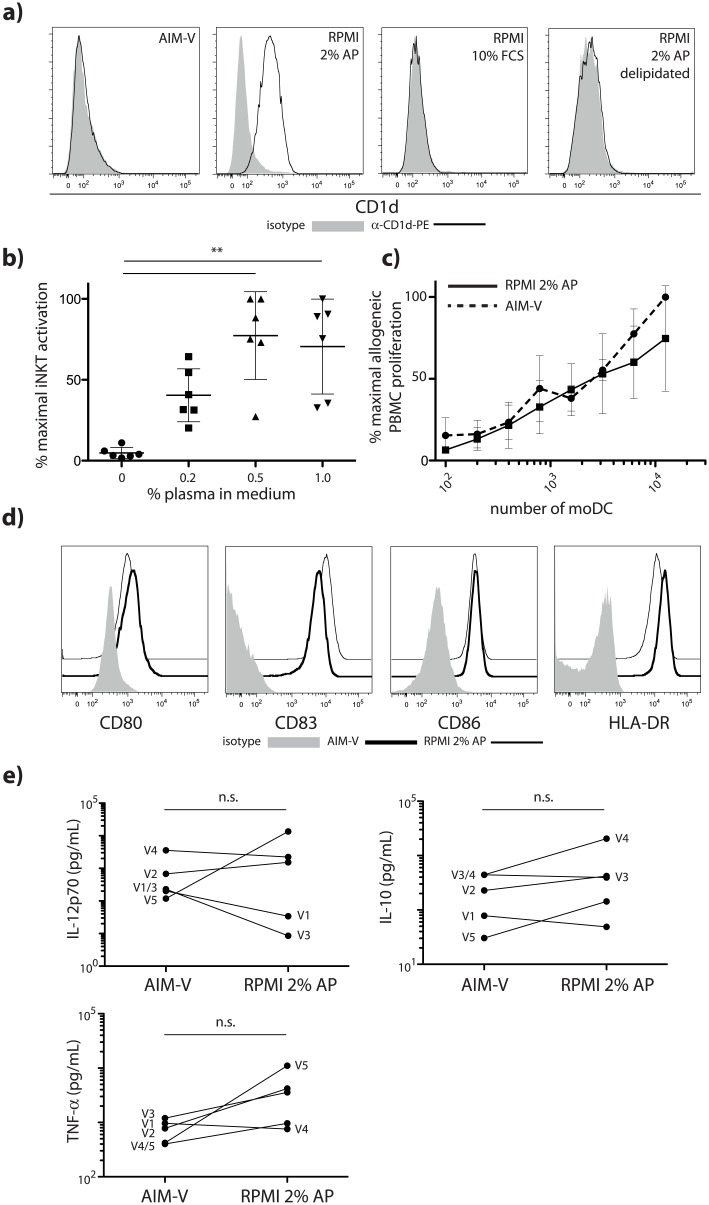
Impact of autologous plasma on expression of CD1d, phenotype and function of moDC. **a)** Representative CD1d expression histograms of moDC cultured in either serum-free AIM-V or RPMI supplemented with either 2% autologous plasma (AP), 10% fetal calf serum (FCS) or 2% delipidated AP. At least three independent experiments were performed. **b)** Activation of NKT hybridoma cells by alpha-GalCer-loaded moDC generated in either AIM-V or RPMI supplemented with 0.2–1% AP. NKT cell activation was assessed using an IL-2 bioassay. Percentages are relative to the highest activation achieved using 1% AP in the culture medium. Mean and error (SD) are shown. ** p<0.01 as tested by one way ANOVA (Kruskal-Wallis test with Dunn’s post test) **c)** Proliferation of allogeneic PBMC in a mixed lymphocyte reaction. 10^5^ PBMC were incubated with increasing numbers of moDC. PBMC proliferation was measured by ^3^H-thymidine uptake. Percentages are relative to the highest cell proliferation observed with 12.5 x 10^3^ moDC^AIM-V^. Mean and error (SD) are shown. **d)** Representative flow cytometry histograms depicting the expression levels of CD80, CD83, CD86 and HLA-DR on moDC matured overnight with IL-1-beta, IL-6, TNF-alpha and PGE_2_. At least three independent experiments were performed. **e)** Cytokine secretion of LPS/IFN-gamma activated moDC as measured by multiplex assay. Each line represents one individual. V1-V5 denominates the five different individuals tested. Statistical significance was determined using a non-parametric Mann-Whitney test.

We examined the requirements for the generation of functional moDC that express CD1d so that and they can be loaded with alpha-GalCer for immunotherapeutic purposes. The use of autologous plasma is common for the generation of clinical grade moDC, although plasma from cancer patients potentially contains immunosuppressive factors that interfere with differentiation into immunogenic cells [12, 13]. We therefore compared cultures generated in autologous plasma-supplemented RPMI (moDC^AP^) to those using the serum-free medium AIM-V (moDC^AIM-V^). In repeated experiments, using cells from different donors, we showed that plasma of human origin was required for the retention of CD1d during the differentiation of monocytes into moDC (Fig 1a). In contrast, moDC^AIM-V^ or moDC prepared in RPMI supplemented with up to 10% serum of bovine origin failed to express detectable amounts of CD1d by flow cytometry (Fig 1a). This observation is consistent with the capacity of human plasma-derived lipoproteins to activate peroxisome proliferator-activated receptor (PPAR)-gamma and sustain CD1d expression through downstream retinoid signaling [7, 14, 15]. Accordingly, the removal of the lipid fraction of plasma resulted in a strong downregulation of CD1d (Fig 1a). Plasma-derived immune complexes have also been shown to contribute to CD1d retention through Fc-gamma-RIIa signaling [6] although delipidation is unlikely to have interfered with this process. The observed plasma-mediated retention of CD1d was dose-dependent, with increasing plasma levels associated with greater capacity of alpha-GalCer-loaded moDC to stimulate iNKT cells (Fig 1b). The addition of plasma did not have any impact on the general immunogenicity of moDC as moDC^AP^ and moDC^AIM-V^ had similar capacities to activate allogeneic T cells (Fig 1c). Furthermore their maturation states after overnight exposure to the established maturation ‘cocktail’ consisting of IL-1-beta, IL-6, TNF-alpha and PGE_2_ were almost identical (Fig 1d), as were their cytokine profiles upon stimulation with LPS and interferon (IFN)-gamma (Fig 1e).

### Impact of autologous plasma on expression of CD1a and CD1a-associated functional dichotomy

**Fig 2:**
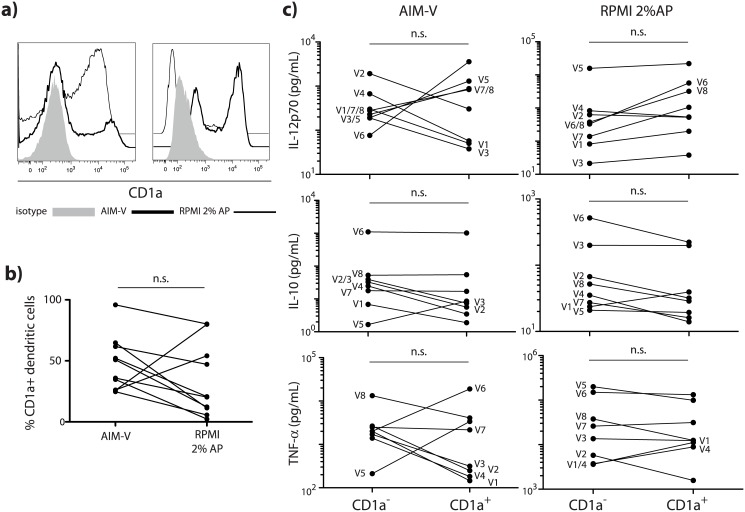
Impact of autologous plasma on expression of CD1a and CD1a-associated functional dichotomy. **a)** CD1a expression histogram plots of moDC generated from two individuals with opposite response patterns to autologous plasma (AP). The left plot depicts moDC that upregulated CD1a in the presence of AP while the individual represented in the right plot downregulated CD1a in response to AP. **b)** Percentages of CD1a^+^ cells in moDC cultures generated in either AIM-V or RPMI 2% AP. Each line represents one individual. Statistical significance was determined using a non-parametric Mann-Whitney test. **c)** Cytokine secretion of LPS/IFN-gamma activated CD1a^-^ and CD1a^+^ moDC as measured by multiplex assay. Each line represents one individual. V1-V8 denominates the eight different individuals tested (V1-V5 identical to Fig 1). Statistical significance was determined using a non-parametric Mann-Whitney test.

The expression of CD1d and CD1a has been shown to be inversely regulated downstream of both retinoid and Fc-gamma—RIIa signaling [6, 7]. However, to our knowledge this association was never tested in the presence of complete human plasma. Intriguingly, while autologous plasma retained expression of CD1d in our experiments, this was not consistently associated with downregulation of CD1a. In fact, in 2 of 10 donors the presence of autologous plasma led to an upregulation of CD1a and an associated increase in CD1a^+^ moDC (Fig 2a and 2b). These results could reflect a large variance among the general population in levels of circulating PPAR-gamma activators such as oxidized low-density lipoprotein [7, 16], or levels of immunoglobulins, which can control the co-expression of CD1a and CD1d [17]. To test whether there was functional dichotomy between CD1a^+^ and CD1a^-^ moDC, we sorted CD1a^+^ and CD1a^-^ cells from moDC^AIM-V^ and moDC^AP^ and assessed their cytokine profile upon stimulation with LPS and IFN-gamma. Surprisingly, we could not observe any consistent functional differences between CD1a^+^ and CD1a^-^ cells regardless of culture conditions (Fig 2c). CD1a^+^ and CD1a^-^ moDC^AP^ differed with similar inconsistency in their ability to activate iNKT cells, notwithstanding comparable CD1d-expression levels (data not shown).

In summary, these data show that the association between the expression of CD1a and type 1 polarized moDC function is not as clear cut as earlier studies had suggested and are more in line with more recent work showing that genetic polymorphism can affect the expression of CD1a without impacting moDC function [18]. Together these observations suggest that, on a population level, CD1a is unlikely to retain a functionally relevant distribution, and the use of CD1a as a surrogate marker for moDC functionality needs critical reexamination. Our results further indicate that the presentation of alpha-GalCer via CD1d can be used as an enhancement to moDC-based vaccines without intrinsic compromise.

## Materials and Methods

### Cells and reagents

Leukocytes and autologous plasma were collected by leukapheresis of health volunteers, with peripheral blood mononuclear cells (PBMC) enriched by density centrifugation using lymphoprep (Axis-Shield, Oslo, Norway) as previously reported [19]. All donors gave informed written consent, and the study was approved by the Central Health and Disability Ethics Committee (LRS/11/03/008/AM03). Cells were cultured in either RPMI-1640 or AIM-V media (Life Technologies, CA, USA) with supplements as indicated in the text. Fetal calf serum was sourced from Life Technologies.

### Dendritic cell culture, phenotype and functional assays

Enriched PBMC were cultured for 5 days in medium supplemented with 1000 IU/ml rhGM-CSF (Bayer, Leverkusen, Germany) and 1000 IU/ml rhIL-4 (Life Technologies) on days 1 and 3. Where indicated, autologous plasma was supplemented up to 2%. In some cases plasma was delipidated before use by organic solvent extraction, as previously described [20]. Briefly, autologous plasma was mixed with 2 volumes of organic solvent composed of butanol and di-isopropylether (40%/60% v/v). The mixture was agitated on a rotator for one hour at room temperature and the delipidated aqueous phase was subsequently harvested with needle and syringe.

The cultures were harvested on day 5, and phenotypic analysis performed by flow cytometry on a LSRII flow cytometer (BD Biosciences, CA, USA). To determine the purity of cultured moDC, the human Mo-DC Differentiation Inspector staining kit from Miltenyi Biotec (Bergisch Gladbach, Germany) was used. The moDC generated according to our protocols were typically >95% CD14^-^CD209^+^. Other antibodies used were against CD1a (FITC-conjugated; clone HI149), CD83 (PerCP-Cy5.5-conjugated; HB15e), CD86 (PE-Cy7-conjugated; IT2.2) and HLA-DR (Alexa-Fluor 700-conjugated; L243), all obtained from Biolegend (CA, USA), and CD1d (PE-conjugated; 51.1) and CD80 (FITC-conjugated; L307), obtained from eBioscience (CA, USA) and BD respectively. For sorting, moDC were labeled with anti-CD1a antibodies and DAPI (Biolegend) and CD1a^+^ and CD1a^-^ fractions collected using an Influx cell sorter (BD Biosciences). For activation, 0.5x10^5^ sorted moDC were stimulated with 100ng/ml LPS (Sigma-Aldrich, MO, USA) and 100ng/ml human IFN-gamma (Peprotech, NJ, USA) for 48h. Cell culture supernatants were analyzed using Bioplex assay kits, following manufacturers instructions (Biorad, CA, USA). The ability of moDC to stimulate iNKT cells was assessed by incubating 25,000 moDC loaded with 100ng/ml alpha-GalCer (kindly provided by Professor Gavin Painter, Victoria University, Wellington, New Zealand) with 10^5^ mouse NKT hybridoma cells (DN32.D3) for 24h and quantifying the secreted IL-2 using an HT-2 bioassay [21]. Briefly, 10^4^ HT-2 cells were seeded in a 96 flat bottom well plate and cultured with 1:1 volume of moDC-DN32 supernatant in RPMI-1640 medium containing 10% fetal calf serum. The following day, ^3^H-thymidine was added to the culture and IL-2-dependent proliferation and ^3^H-thymidine uptake were quantified using a Microbeta-counter (Perkin-Elmer, MA, USA). Similarly, to assess immunogenicity, mixed lymphocyte reactions were performed with titrated numbers of moDC cultured with 1x10^5^ allogeneic PBMC for 72h. Proliferation of responders was assessed by examining ^3^H-thymidine uptake for the last 18h of culture.
